# *Melissa officinalis* extract suppresses endoplasmic reticulum stress-induced apoptosis in the brain of hypothyroidism-induced rats exposed to γ-radiation

**DOI:** 10.1007/s12192-023-01363-8

**Published:** 2023-06-27

**Authors:** Omayma AR Abo-Zaid, Fatma SM Moawed, Eman FS Taha, Esraa S.A. Ahmed, Ragaa SM Kawara

**Affiliations:** 1 Biochemistry and Molecular Biology Department, Faculty of Vet. Med, Benha University, Moshtohor, Banha, Egypt; 2 Health Radiation Research, National Center for Radiation Research and Technology, Egyptian Atomic Energy Authority, Cairo, Egypt; 3 Radiation Biology Research, National Center for Radiation Research and Technology, Egyptian Atomic Energy Authority, Cairo, Egypt; 4 Egyptian Atomic Energy Authority, Nasr City, Cairo, 11787 Egypt

**Keywords:** Hypothyroidism, *Melissa officinalis*, γ-radiation, Brain damage, Endoplasmic reticulum stress, ERAD

## Abstract

The purpose of this study was to demonstrate the neuroprotective effect of *Melissa officinalis* extract (MEE) against brain damage associated with hypothyroidism induced by propylthiouracil (PTU) and/or γ-radiation (IR) in rats. Hypothyroidism induction and/or exposure to IR resulted in a significant decrease in the serum levels of T3 and T4 associated with increased levels of lipid peroxidation end product, malondialdehyde (MDA), and nitrites (NO) in the brain tissue homogenate. Also, hypothyroidism and /or exposure to IR markedly enhance the endoplasmic reticulum stress by upregulating the gene expressions of the protein kinase RNA-like endoplasmic reticulum kinase (PERK), activated transcription factor 6 (ATF6), endoplasmic reticulum-associated degradation (ERAD), and CCAAT/enhancer-binding protein homologous protein (CHOP) in the brain tissue homogenate associated with a proapoptotic state which indicated by the overexpression of Bax, BCl2, and caspase-12 that culminates in brain damage. Meanwhile, the PTU and /or IR-exposed rats treated with MEE reduced oxidative stress and ERAD through ATF6. Also, the MEE treatment prevented the Bax and caspase-12 gene expression from increasing. This treatment in hypothyroid animals was associated with neuronal protection as indicated by the downregulation in the gene expressions of the microtubule-associated protein tau (MAPT) and amyloid precursor protein (APP) in the brain tissue. Furthermore, the administration of MEE ameliorates the histological structure of brain tissue. In conclusion, MEE might prevent hypothyroidism-induced brain damage associated with oxidative stress and endoplasmic reticulum stress.

## Introduction

Hypothyroidism occurs when the thyroid gland fails to produce enough thyroid hormone to meet the body’s metabolic demands. In combination with low levels of thyroxine (T4) and triiodothyronine (T3), hypothyroidism produces elevated levels of serum thyrotropin-stimulating hormone (TSH) (Muppidi et al. 2023). The thyroid hormone is essential to energy metabolism, mitochondrial function, oxygen consumption, and active oxygen metabolism (De Vitis et al. 2022; Cioffi et al. 2022). Moreover, thyroid hormones have a vital role in normal brain development and function throughout life. They are required for growth, neurogenesis, neuronal differentiation, and metabolic regulation (Chaker et al. 2022). However, a deficiency of thyroid hormone during brain development (fetal and postnatal) retarded maturation, impaired cognitive and neurological functions, and finally neuronal death (Liu and Brent 2018) due to the sensitivity of the hippocampus to thyroid hormone (Alva-Sánchez et al. 2009). It was found that people suffering from thyroid hormone deficiency exhibit a wide range of clinical symptoms, including neurological impairment, difficulty in memory and concentration, and depression (Samuels and Bernstein 2022). Experimentally induced hypothyroidism resulted in growth and structural abnormalities of the neurons in the cortex and hippocampus, and impaired neurological function of neonatal rats (Talhada et al. 2019; Hashem and Saad 2020). There is a connection between hypothyroidism and a decrease in long-term potentiation (Głombik et al. 2021) and deterioration of memory and learning capacity (Alzoubi et al. 2009). Hypothyroidism causes an increase in 8-hydroxyguanosine, which is accompanied by an increase in caspase-3 in serum and brain tissues, indicating an oxidative stress state leading to an increase in the percentage of DNA damage in the brain (Wahman et al. 2020).

Radiation therapy has been identified as the cause of several thyroid disorders, including hypothyroidism, thyroiditis, Graves’ disease, adenoma, and carcinoma (Nagayama 2018). Exposure of tissues to radiation stimulates water hydrolysis generating reactive oxygen species (ROS) which damage cellular components (DNA, lipids, and proteins) together with impairing the cellular organelles (mitochondria and endoplasmic reticulum (ER)) and signalling pathways (Cervelli et al. 2021; Abo-Zaid et al. 2023). The ER is a membranous organelle which dominates various cellular processes like protein synthesis and folding, lipid synthesis, and calcium homeostasis (Phillips and Voeltz 2016). The impaired redox status coupled with ROS perturbed the ER homeostasis and function (disrupted protein folding process) causing the accumulation of misfolded proteins which exceed the capacity of ER chaperones and concomitantly provoke ER stress. Consequently, stimulation of the unfolded protein response (UPR) signalling pathway in the lumen of the ER to restore homeostasis (Hetz and Papa 2018) subsequent to the activation of ER transmembrane receptors: pancreatic ER kinase (PKR)-like ER kinase (PERK) which hindered protein synthesis and translation by phosphorylating eukaryotic initiation factor 2α (eIF2α) followed by the activating transcription factor 6 (ATF6). Additionally, the activated UPR pathway provoked the removal and degradation of accumulated misfolded proteins by ER-associated protein degradation (ERAD), enhancement of protein folding by the molecular chaperones, and induction of autophagy (Chen and Cubillos-Ruiz 2021).

However, the persistent state of ER stress overwhelmed the ability of the UPR to retrieve the ER homeostasis thus inducing apoptosis and cell death (Sandow et al. 2014) via activation of the pro-apoptotic CCAAT/enhancer-binding protein-homologous protein (CHOP) (Mao et al. 2019). CHOP mediates ER stress-induced apoptosis through modulation of the downstream pro-apoptotic proteins (BCL2 family) and caspases particularly caspase-12 which is found in the ER outer membrane and promoted ER stress-mediated apoptosis by activation of the mitochondrial caspases (3 &9) (Han et al. 2021).

Natural plant products have proven to be effective neuroprotective molecules in the treatment of neurological diseases. *Melissa officinalis*, known as lemon balm, is an old natural medicinal plant commonly consumed as herbal tea to ameliorate digestion and gastrointestinal disorders due to its antispasmodic properties (Sipos et al. 2021). The *Melissa officinalis* ethanolic extract (MEE) contains various phytochemicals compounds, including flavonoids, rosmaric acid, gallic acid, and phenolic contents, which confer its therapeutic potential as antioxidant, antiviral, anti-inflammatory, neuroprotective, and anticarcinogen (Kamdem et al. 2013). Consequently, a previous study reported the protective effect of the MEE against oxidative stress-derived degenerative diseases (Sipos et al. 2021). Moreover, MEE has been shown to improve short-term memory and learning in patients (Abdel-Aziz 2018). Thus, the purpose of this study was to evaluate the neuroprotective mechanism of *MEE* associated with oxidative stress and endoplasmic reticulum stresses in the brain of propylthiouracil (PTU) and/or γ-radiation (IR)-induced-hypothyroidism in rats.

## Materials and methods

### Plant materials

*Melissa officinalis*, an aromatic herb of the mint family (Lamiaceae), was obtained from Abd El-Rahman Harraz (Bab El-Khalk Zone, Cairo, Egypt). A plant was identified at Al-Azahr University, Faculty of Science, (Boys) by Abdel-Aziz (2018), who reported that *M. officinalis* contains high amounts of flavonoids, rosmaric acid, gallic acid, and phenolic contents (Miraj and Rafieian-Kopaei 2017).

### Melissa officinalis extraction

For the extraction process, the herbal leaves were air-dried, ground, and immersed in ethanol (1:10 w/v) for 3 days while being continuously shaken. Following filtration, the solvent was expelled using a rotary evaporator at low pressure until dryness was attained. The yield percentage was then determined as 1 g (extract)/100 g. (crude powdered herb). The *Melissa officinalis* ethanolic extract (MEE) was kept at −20 °C until use according to the method described by Mannaa et al. (2021). Rats were given 75 mg/kg/day of MEE extract orally at the beginning of the third week of the PTU injection and continued for 2 weeks (14 days) (Abdel-Aziz 2018).

### Chemicals

Propylthiouracil (PTU) was obtained from Sigma Aldrich Chemical Co. (St. Louis, USA). All chemicals were of analytical grade.

### Irradiation process

At the National Center for Radiation Research and Technology (NCRRT, Cairo, Egypt), whole-body gamma irradiation was performed using Canadian gamma cell-40 (137Cesium) at a dose rate of 0.333 Gy•min^−1^ for a total dose of 5 Gy in a single dose on the 21st day after the last dose of the PTU (Ebrahim 2020).

### Animals

In the current investigation, 36 Wister male albino rats weighing 120–150 g with an average age of 3 months were employed. Animals were obtained for pharmaceutical purposes from the Nile Company”s breeding unit in Egypt. Rats were housed in the laboratory room for at least 1 week before testing. They were kept under standard conditions of humidity (50±5%) and subjected to a 12:12-h light-dark cycle. Animals were maintained on starter poultry pellets and water ad libitum for 1 week before starting the experiment as an acclimatization period. The treatment of the experimental animals was consistent with the guidelines of ethics in the Guide for the Care and Use of Laboratory Animals (1997), in accordance with the recommendations of the animal care committee of the National Center for Radiation Research and Technology (NCRRT), Cairo, Egypt.

### Induction of hypothyroidism

Hypothyroidism was induced according to the method of Issa and El-Sherif (2017) with minor modifications by a daily i.p. injection of PTU (5 mg/kg body weight) for 3 weeks.

### Experimental groups

After an acclimatization period of 1 week, rats were divided into six groups (*n* = 6) as follows: Control group: Normal rats were intraperitoneally (i.p) given 0.5 ml physiological saline daily for 30 days. PTU group: rats were injected i.p. with PTU (5 mg/kg/day for 21 days). PTU+IR group: rats were injected with PTU (5 mg/kg/day for 21 days) and were exposed to a single whole-body gamma IR at a dose of 5 Gy on the 21st day. MEE group: rats were given 75 mg/kg/day MEE orally at the beginning of the third week of the PTU injection and continued for 2 weeks (14 days) (Abdel-Aziz 2018). PTU+MEE group: rats were injected with PTU (5 mg/kg/day for 21 days) and were treated with MEE. PTU+IR+MEE group: rats were injected with PTU, exposed to IR, and treated with MEE as mentioned above.

**Fig. 1:**
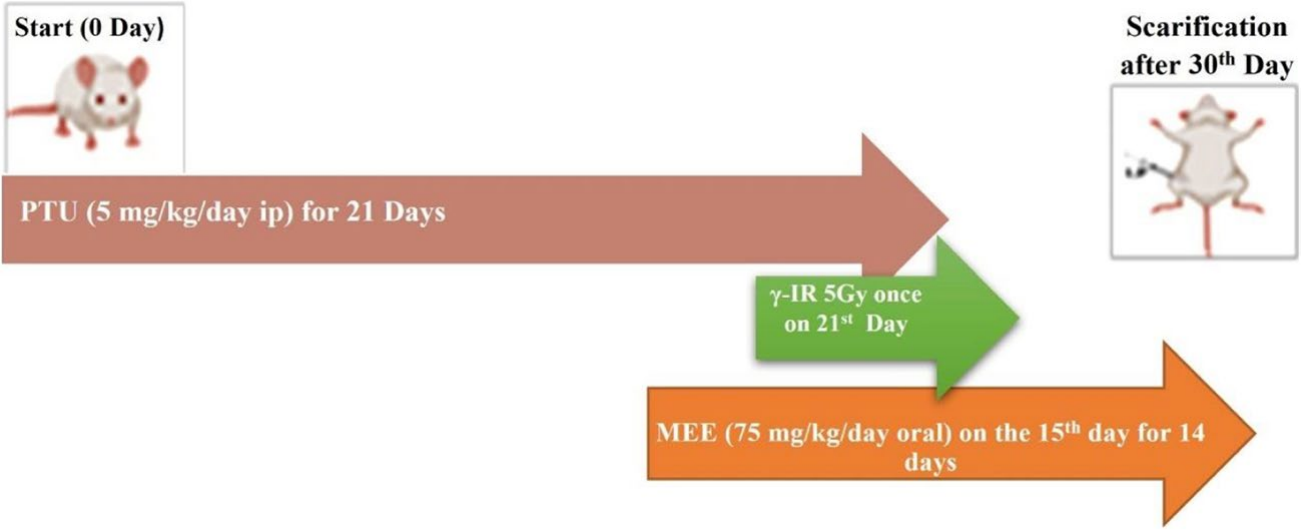
Experimental group design

At the end of the experimental period, the animals were fasted overnight and sacrificed under anesthesia using urethane. Blood samples were collected from each rat and were centrifuged to obtain serum. The brain was dissected out and washed with saline, dried on filter paper, and then a part of each brain was weighed and homogenized in a 50 mmol/l phosphate buffer (ice cold) solution (pH 7.4) to give 10% w/v homogenate. The homogenate was centrifuged at 3000 rpm for 15 min at 4 °C. The clear supernatant was separated for further determination of the biochemical parameters. Moreover, the other part of the brain was dissected and immediately kept in a 10% buffered formalin-saline solution for a later histopathological examination (Fig. 1).

### Histopathological examination

Brain tissue samples were collected and fixed in a 10% neutral buffered formalin solution for histopathology. Tissue specimens were processed as follows: dehydrated in an ascending concentration of ethanol, cleared in xylene, embedded in paraffin wax, and sectioned at a 5-μm thickness (Bancroft and Gamble 2013). Prepared slide sections were stained with hematoxylin and eosin and examined by a light digital microscope (Olympus XC30, Tokyo, Japan). They were then scored using the following histological grading system (Kato et al. 1991): I, a few neurons damaged; II, numerous neurons damaged; III, the majority of neurons damaged; IV, the vast majority of neurons damaged.

### Biochemical determinations

Serum thyroid-stimulating hormone (TSH; No. MBS701641), total triiodothyronine (T3; No MBS261285), and total thyroxine (T4; No MBS704309) levels were determined by enzyme-linked immunosorbent assay (ELISA) using rat ELISA kits purchased from MyBioSource Co. (San Diego, California, USA). Malondialdehyde content (MDA; No MD2529), the activity of glutathione peroxidase (GPx; No GP 2524), level of glutathione reduced (GSH; No GR2511), catalase activity (CAT; No. CA 2517), superoxide dismutase activity (SOD; No SD 2521), and nitric oxide level (NO; No NO2533) in the supernatant of brain homogenates were measured calorimetrically using a commercial kit (Bio-diagnostic, Egypt).

### Analysis of gene expression by real-time quantitative reverse transcription–polymerase chain reaction (RT-qPCR)

Pure RNA was extracted using the total RNA purification kit (Thermo Scientific, Ferments, #K0731), and cDNAs were synthesized using a RevertAid First Strand cDNA Synthesis Kit and reverse transcription kits (Thermo Scientific, Ferments, # EP0451) following the manufacturer's instructions. Gene expression was measured using RT-PCR with SYBR Green. The isolated cDNA was amplified using 2× Maxima SYBR Green/ROX qPCR Master Mix and gene-specific primers, as directed by the manufacturer (Thermo Scientific, USA, # K0221). The primers used in the amplification are shown in Table 1. The web-based tool Primer 3 was used to design these primers based on published rat sequences. To ensure the primer sequence is unique for the template sequence, we checked the similarity to other known sequences with BLAST (www.ncbi.nlm.nih.gov/blast/Blast. cgi). Thermal cycling conditions were 95 °C for 10 min, 95 °C for 15 s, 60 °C for 30 s, and 72 °C for 30 s for 40 cycles. The relative amount of mRNA of each gene in each sample was calculated using the 2^−ΔΔCT^ method (Livak and Schmittgen 2001). All qRT-PCR experiments used actin expression as an internal control.

**Table 1:** Sequences for primers used for quantitative RT-PCR analysis

Gene	Forward primer (5′ ------ 3′)	Reverse primer (5′ ------ 3′)	Accession number
Bax	ACACCTGAGCTGACCTTG	AGCCCATGATGGTTCTGATC	XM_032893644
*Bcl2*	ATCGCTCTGTGGATGACTGAGTAC	AGAGACAGCCAGGAGAAATCAAAC	NM_016993.2
*MAPT*	TGGCTTAAAAGCTGAAGAAGCA	GCCCTTGGCTTTCTTCTCGT	NM_017212.3
*APP*	CAACCGTGGCATCCTTTTGG	CGTCGACAGGCTCAACTTCA	XM_032900495
*CHOP*	ACCACCACACCTGAAAGCAG	AGCTGGACACTGTCTCAAAG	XM_039078588
*PERK*	GAAGTGGCAAGAGGAGATGG	GAGTGGCCAGTCTGTGCTTT	NM_031599
*EIF2α*	TTGAACTGTTGTGACCCCGAC	CGTAGTCTGCCCGATTTTGC	
*ATF6*	GGACCAGGTGGTGTCAGAG	GACAGCTCTGCGCTTTGGG	XM_008769738
*ERAD (BiP)*	TAACAATCAAGGTCTACGAAGG	CCATTCACATCTATCTCAAAGGT	*M14050*
*Caspase-12*	TGGATACTCAGTGGTGATAA	ACGGCCAGCAAACTTCATTA	NM_130422
*B-actin*	AAGTCCCTCACCCTCCCAAAAG	AAGCAATGCTGTCACCTTCCC	XM_032887061

*PERK*, protein kinase-like endoplasmic reticulum kinase; *ATF-6*, activating transcription factor 6; *CHOP*, CCAAT/enhancer-binding protein homologous protein; *eIF2α*, eukaryotic translation initiation factor 2A; *Caspase-12*, cysteinyl aspartate specific proteinase 12; *BAX*, BCL2 associated X, apoptosis regulator; *Bcl-2*, B-cell lymphoma 2; *MAPT*, microtubule-associated protein tau; *APP*, amyloid precursor protein; *ERAD*, endoplasmic reticulum-associated degradation

### Statistical analysis

Using the SPSS 20 software package (Analytical Software, USA), the results were statistically analyzed by one-way ANOVA, followed by Duncan’s test to assess the differences between groups at a *p*<0.05. The data are expressed as mean ± SE (*n* = 6 per group). Moreover, the charts were graphed via GraphPad Prism 8 (GraphPad, CA, USA).

## Results

### Histopathological examination

**Fig. 2:**
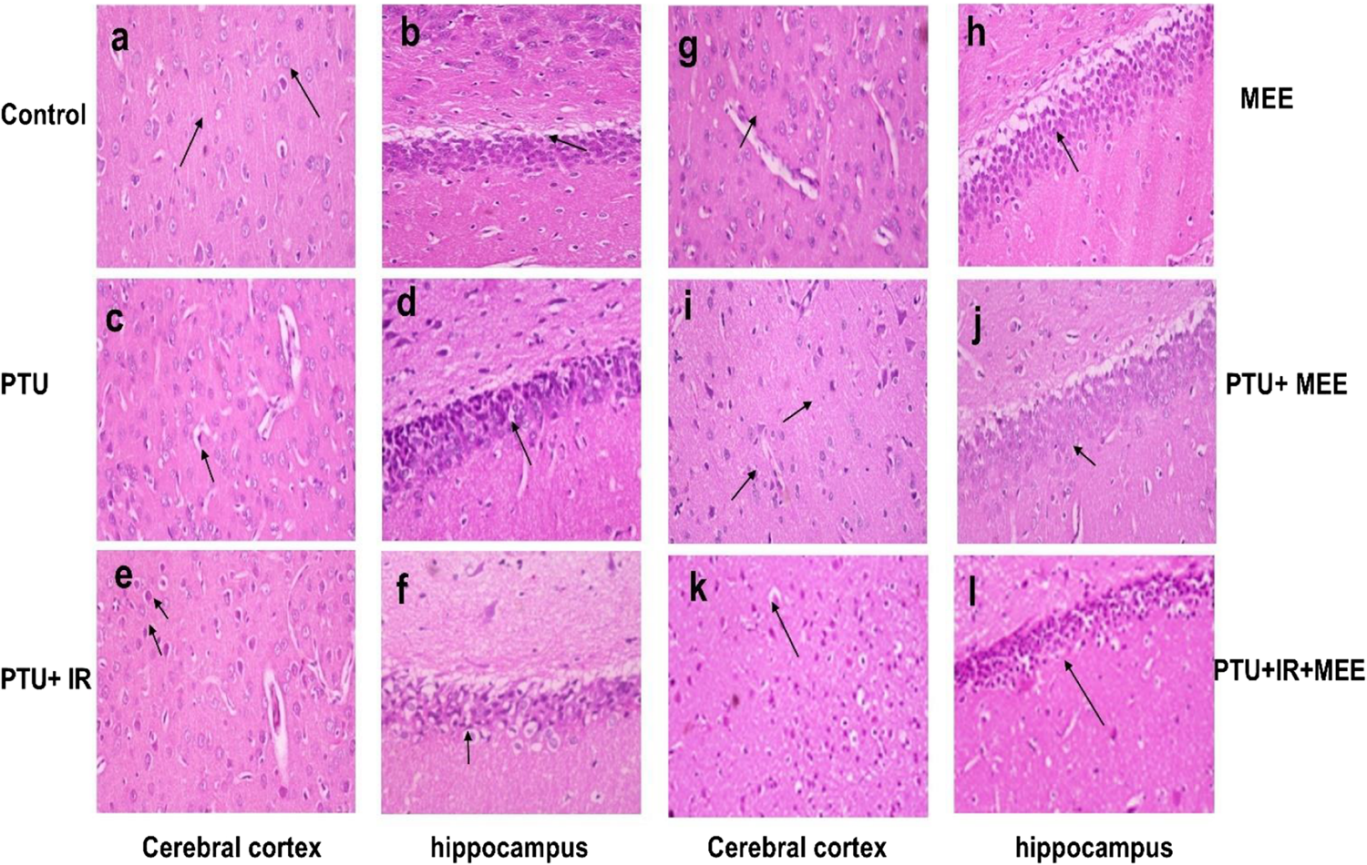
Photomicrograph of brain tissue section showing (**a**, **g**) normal histological structure of the cerebral cortex, (**b**, **h**) normal granular cell layers of the dentate gyrus arrow, (**c**) degenerated and apoptotic neuronal cells surrounded by per-cellular haloes arrow, (**d**) vacuolar degeneration of the granular cell layers with dark nuclei arrow, (**e**) apoptosis of neuronal cells which appeared as densely eosinophilic bodies, (**f**) vacuolar degeneration of granular cells arrow, (**i**) few numbers of degenerated neuronal cells arrows, (**j**) normal granular cell layers, (**k**) low neuronal degeneration, and (**l**) normal organization of the compact granular cell layer arrow (H&E×400)

The cerebral cortex tissue section from the control and MEE group showed normal histopathological structure consisting of many layers of neuronal cells having an oval-rounded nuclei surrounded by scanty basophilic cytoplasm and small blood vessels in between (score 0) (Fig. 2a and g), while the hippocampal section (dentate gyrus region) exhibited normal granular layer (cells with dark nuclei), molecular layer (glial cells as well as pyramidal cells) (score 0) (Fig. 2b and h). However, the cerebral cortex of rats injected with PTU exhibited moderate neuronal degeneration in the form of shrunken and darkly pyknotic nuclei. Neuronal cell apoptosis manifested as densely eosinophilic bodies (score 2) (Fig. 2c). Histological sections of the hippocampal (dentate gyrus region) revealed pyramidal cell shrinkage and vacuolar degeneration of the granular cell layers (score 2) (Fig. 2d). Additionally, the cerebral cortex of the PTU+ IR group showed neuronal degeneration manifested as darkly stained condensed and clumped nuclear chromatin surrounded by per-cellular halo spaces accompanied by focal gliosis, neuronophagia with perivascular edema, and apoptotic neuronal cells (score 3) (Fig. 2e), while the histological section of the hippocampal (dentate gyrus region) revealed cellular disorganization and marked shrinkage in the size of large pyramidal cells together with remarkable vacuolation of the granular cell layers (score 2) (Fig. 2f). In contrast, treatment with MEE not only lowered the number of degenerated neuronal cells but also alleviated gliosis, satellitosis, neuronophagia, and perivascular edema of the cerebral cortex (score 1) (Fig. 2i and k) besides restoring the structure of the granular cell layers of the hippocampal dentate gyrus region (score 0) (Fig 2j and l) in both PTU+ MEE and PTU+IR+MEE group.

## Biochemical results

### Effect of MEE on thyroid function test (TSH, T3, and T4)

**Fig. 3:**
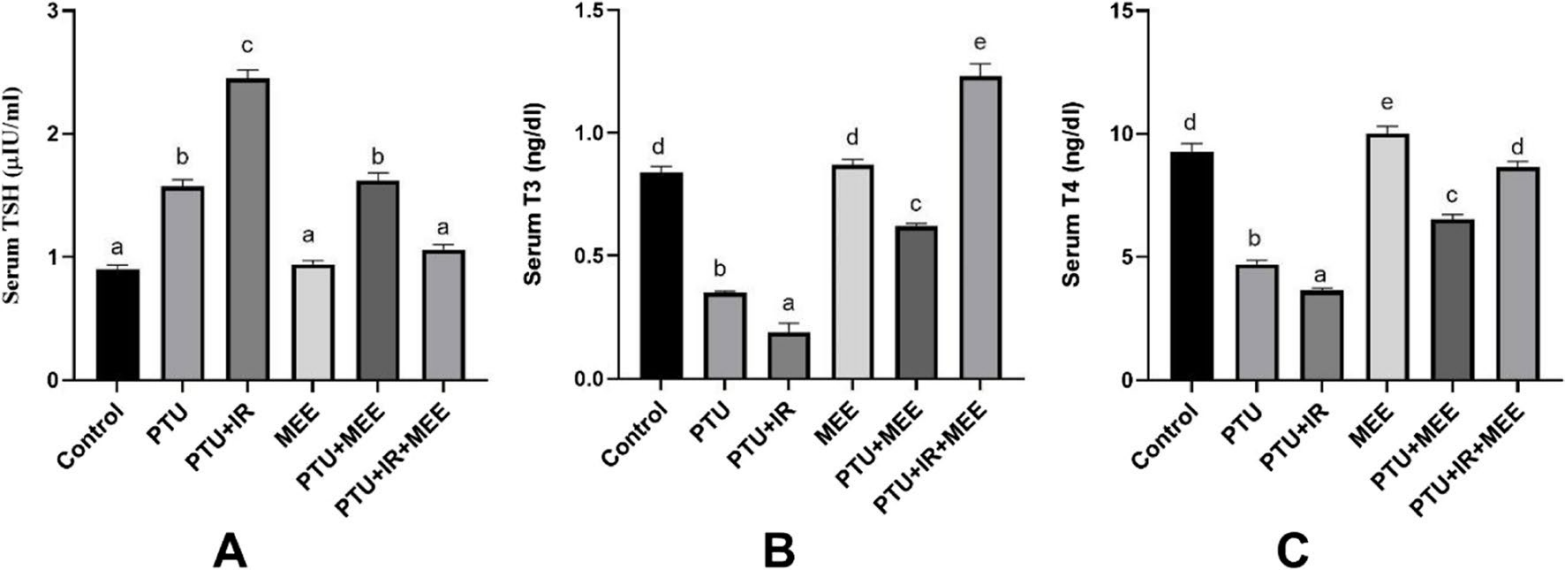
Effect of MEE on serum thyroid function in different experimental groups. (**A**) The levels of TSH. (**B**) The levels of T3. (**C**) The levels of T4 hormone. Values with different symbols are significantly different from each other at *p* < 0.05 while those with similar symbols are non-significant

As shown in Fig. 3, injection of rats with PTU resulted in a significant decrease in serum T3 and T4 levels (−58.35% and −48.9%, respectively) and an increase in TSH (74.4%) as compared to their respective control values, confirming the development of hypothyroidism. However, more disturbance in the thyroid function (T3, T4, and TSH) was observed in the group of rats injected with PTU and exposed to IR (−45.71%, −23.40%, and 56.05% respectively) compared to the control group. On the other hand, administration of MEE significantly improved the thyroid function in both PTU and PTU+IR groups (T3: 77.14, 547.3%; T4: 38.72, 138.29%; and TSH: −23.56, −56.73% respectively) compared with PTU and PTU+IR groups.

### Effect of MEE on brain oxidative stress markers

**Fig. 4:**
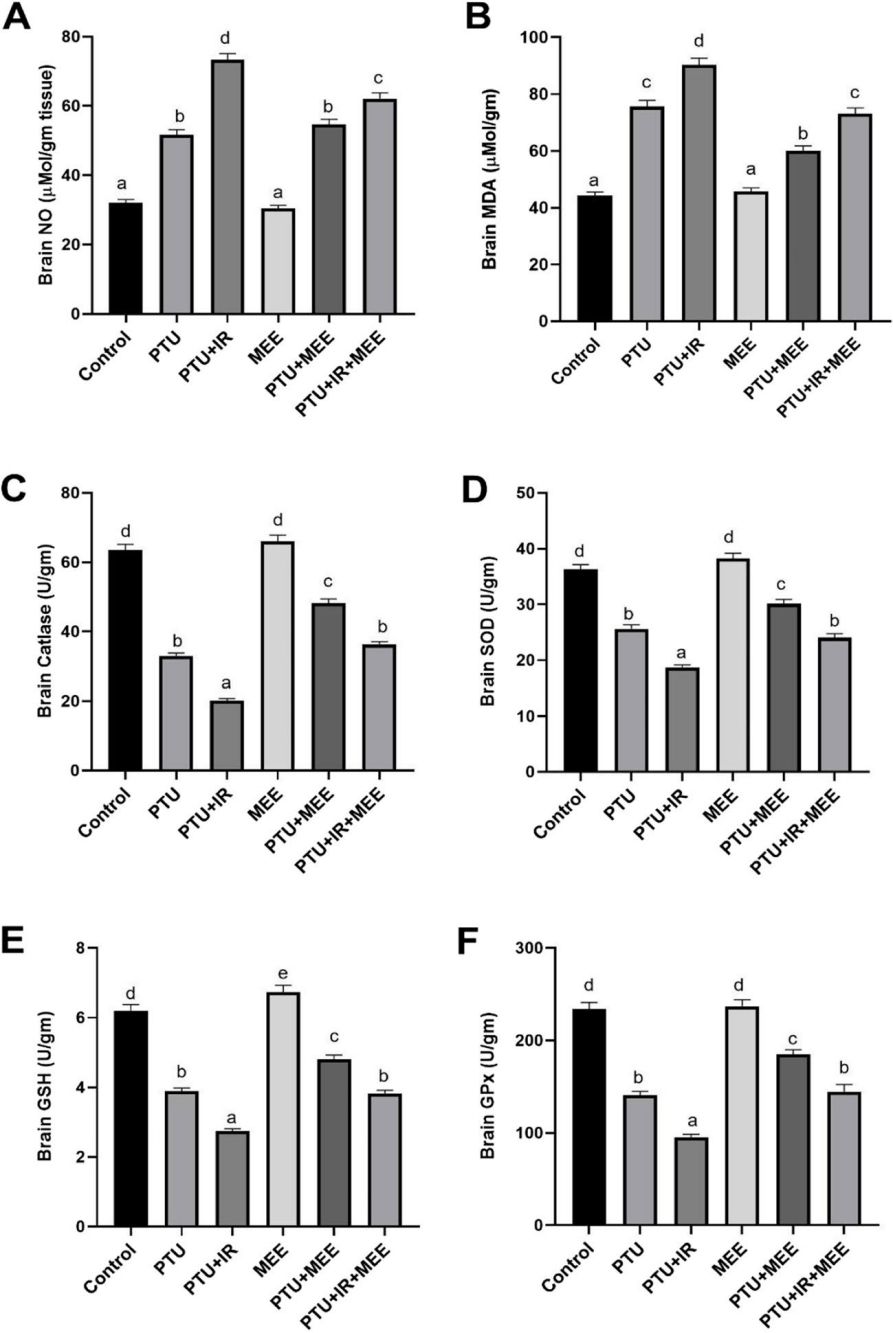
Effect of MEE on hypothyroidism-induced oxidative stress in the brain of rats. (**A**) The levels of NO. (**B**) The levels of MDA. (**C**) The activity of catalase enzyme. (**D**) The activity of SOD. (**E**) The content of GSH. (**F**) The activity of GPx. Values with different symbols are significantly different from each other at *p* < 0.05 while those with similar symbols are non-significant

The activity of different brain antioxidant enzymes (GSH, GPx, SOD, and catalase) was evaluated in irradiated and unirradiated PTU hypothyroidism-induced rats. It was found that both PTU and PTU+IR groups showed a remarkable decrease in GSH levels and activities of GPx, SOD, and catalase in brain tissues accompanied by significant elevation in MDA and NO levels compared with the control group. The excessive impairment in the redox status was observed in the PTU+IR group. The hypothyroidism rats that received MEE showed a notable decline in the brain MDA and NO levels coupled with significant enhancement in the levels of GSH and activities of GPx, SOD, and catalase as compared with the PTU and PTU+IR groups (Fig. 4).

### Effect of MEE on brain markers (MAPT and APP)

**Fig. 5:**
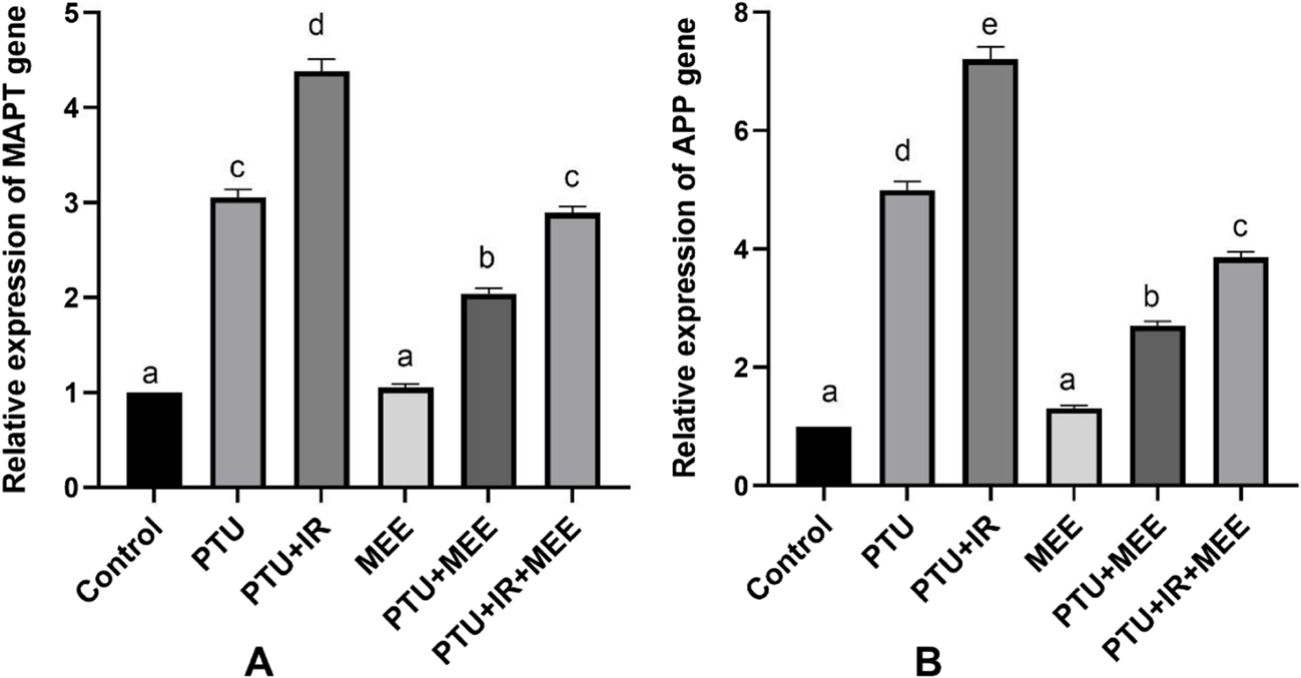
Effect of MEE on brain markers. (**A**) Microtubule-associated protein tau (MAPT) and (**B**) β-amyloid precursor protein (APP), gene expression. Values with different symbols are significantly different from each other at *p* < 0.05 while those with similar symbols are non-significant

Neurodegenerative disease and brain damage are commonly characterized by the accumulation of tau protein and amyloid-β. Therefore, the mRNA expression of the amyloid precursor protein (APP) and microtubules-associated protein tau (MAPT) was examined in the brain tissues to evaluate the damaging effect associated with hypothyroidism. The obtained results in Fig. 5 revealed that hypothyroidism induced either by PTU alone or with gamma IR was associated with remarkable upregulation in the APP and MAPT mRNA expression relative to the control group (*p*<0.001). Conversely, MEE treatment alleviated brain damage by reducing the expression of these genes.

### Effect of MEE on endoplasmic reticulum stress markers (PERK, eIF2α, ATF6, and ERAD) in the brain

**Fig. 6:**
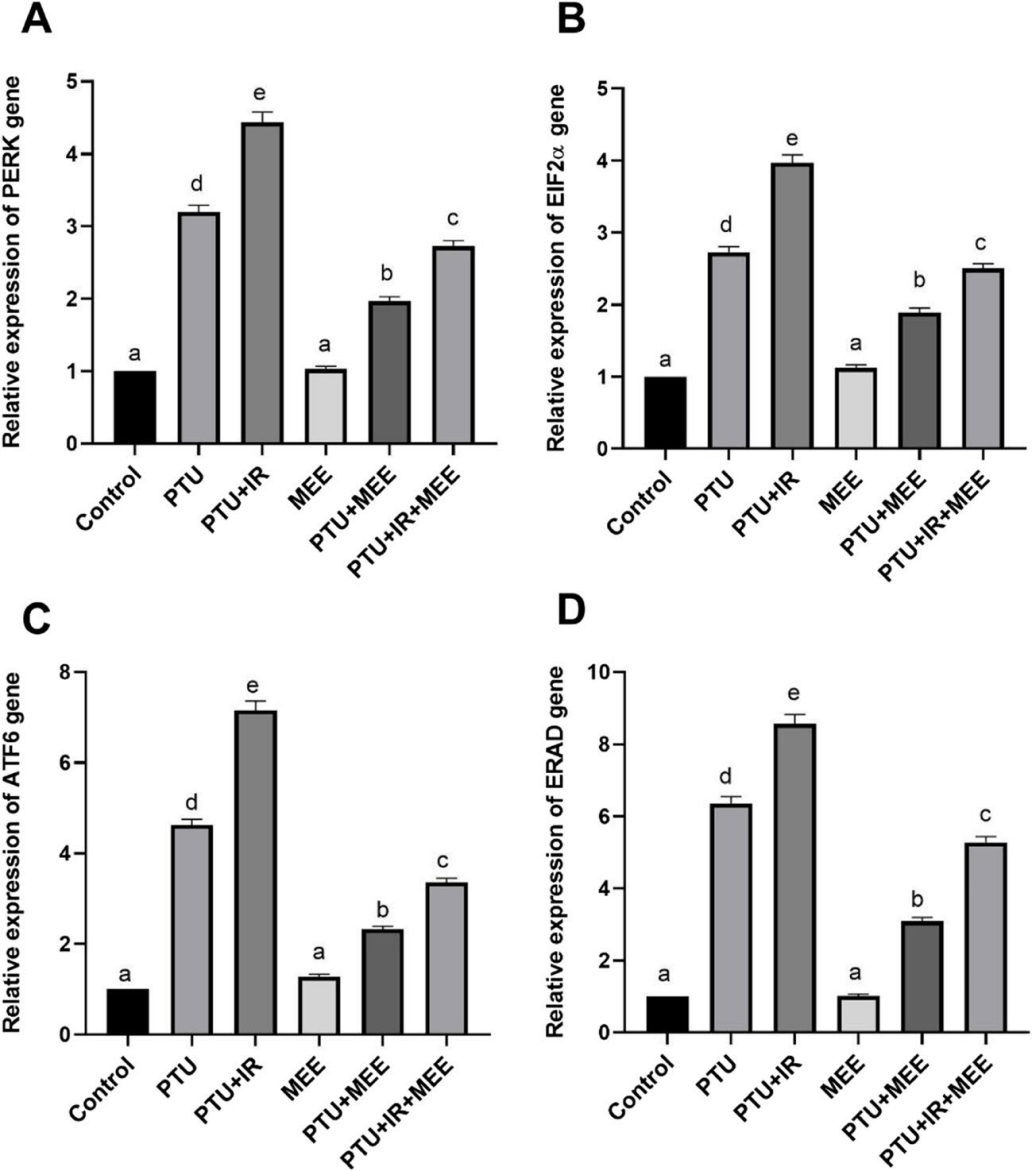
Effect of MEE on endoplasmic reticulum stress biomarkers in the brain tissues. (**A**) The relative expression of PERK. (**B**) The relative expression of eLF2α. (**C**) The relative expression of ATF6. (**D**) The relative expression of ERAD. Values with different symbols are significantly different from each other at *p* < 0.05 while those with similar symbols are non-significant

The evaluation of the endoplasmic reticulum stress biomarkers (PERK-eIF2α-ATF6-ERAD) was shown in Fig. 6. It was observed that PTU injection upregulated the brain expression of PERK (220%), eIF2α (173%), ATF6 (363%), and ERAD (536%). Furthermore, exposure of the rat to gamma radiation after injection with PTU notably caused the overexpression of the ER stress markers (PERK: 344%, eIF2α: 290%, ATF6: 616%, and ERAD: 757%) compared to the control (*p*<0.001). Meanwhile, treating the hypothyroidism animals (both PTU and PTU+IR) with MEE markedly downregulated the expression of these biomarkers compared with PTU and PTU+IR groups respectively.

### Effect of MEE on endoplasmic reticulum stress-induced apoptosis

**Fig. 7:**
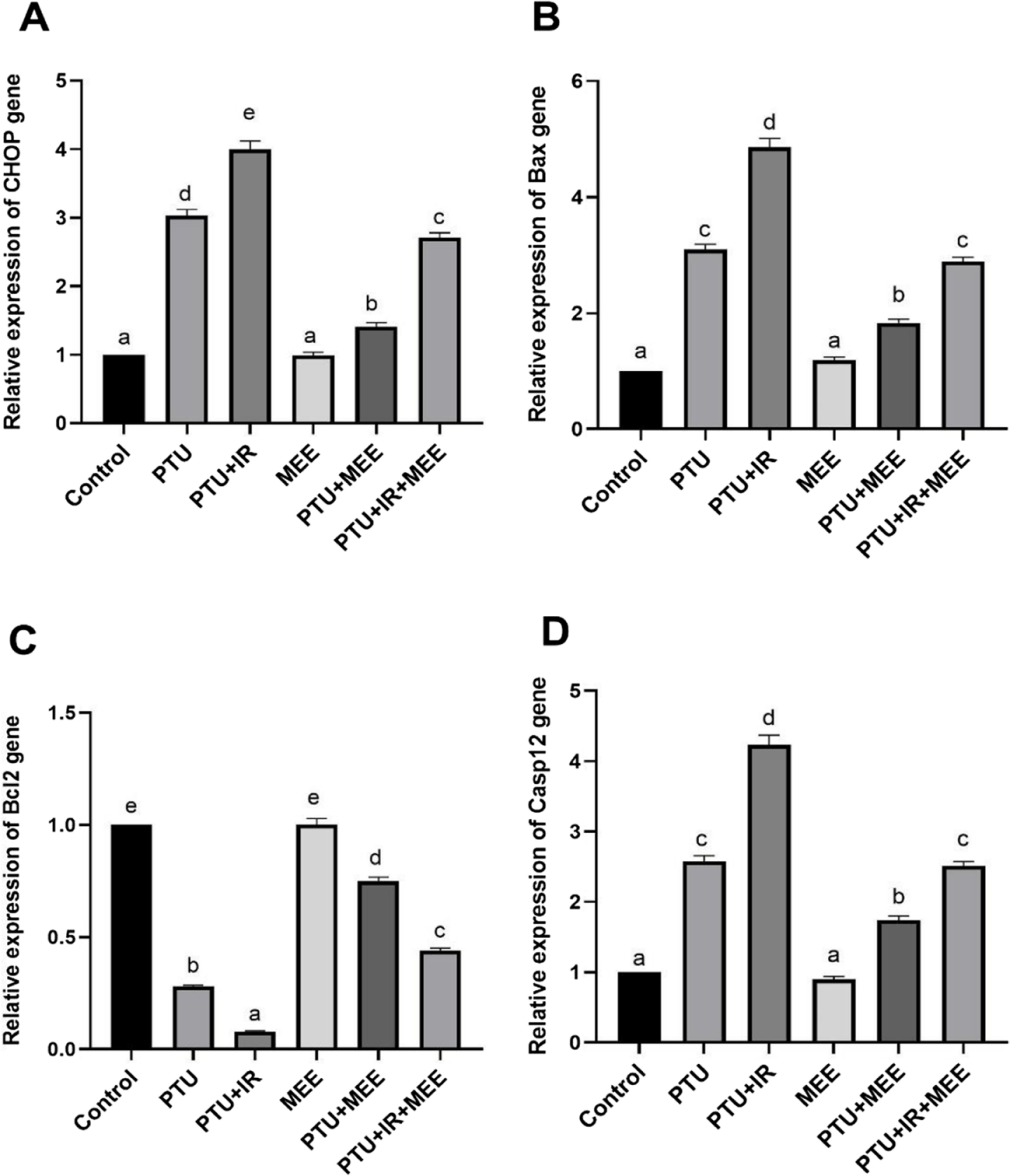
Effect of MEE on the endoplasmic reticulum stress and apoptotic markers in adult rats’ brains. (**A**) The relative expression of CHOP. (**B**) The relative expression of Bax. (**C**) The relative expression of Bcl-2. (**D**) The relative expression of caspase-12. Values with different symbols are significantly different from each other at *p* < 0.05 while those with similar symbols are non-significant

The effect of MEE on the neuronal death markers and apoptotic pathway caused by hypothyroidism in the brains was illustrated in Fig. 7. Hypothyroidism caused overexpression of CHOP (163.3% and 300%), Casp12 (158% and 323%), and Bax (210% and 386%), while downexpression of Bcl2 levels was observed in both the PTU and PTU+IR groups, respectively (*p*<0.001). Whereas, MEE administration significantly downregulated the expression of CHOP (53.46% and 32.25%), Casp12 (32.55% and 40.66%), and Bax (40.96% and 40.53%) in the brain of hypothyroid rats (*p*<0.001). This downregulation was associated with upregulation in the gene expression of Bcl2 (167.85% and 450%) in both non-irradiated and irradiated hypothyroid-induced rats (*p*<0.001).

## Discussion

Several mechanisms of neural damage are associated with PTU-induced hypothyroidism, including oxidative stress, and endoplasmic reticulum stress (Torres-Manzo et al. 2018). The development of a treatment for this disease is still crucial to avoiding neuronal damage. A variety of natural products have been reported to have applications in treating oxidative stress-induced damage in different diseases. In addition to its antioxidant properties, MEE is anti-inflammatory and neuroprotective. The role of MEE in preventing endoplasmic reticulum stress has yet to be determined. As a result, the goal of this study was to determine whether MEE maintains oxidative and endoplasmic reticulum stress in the brains of hypothyroid rats after radiation exposure.

The most effective test for detecting hypothyroidism is the laboratory measurement of serum TSH, T3, and T4 (Mincer and Jialal 2022), and an increase in TSH and a decrease in T4 with normal or decreased T3 levels are regarded as evidence of hypothyroidism (Ashraf et al. 2022). In the current study, injection of the PTU notably elevated the levels of the TSH coupled with a significant decline in the thyroid hormone (T3 and T4) levels relative to their levels in the control rats, ascertaining the hypothyroidism state. These results are in line with the previous studies, which indicated the suppressive effect of the PTU on the thyroid peroxidase as well as type I deiodinase enzymes that are responsible for the synthesis of T4 and its conversion to T3 respectively leading to their reduction in serum (Fumarola et al. 2010; Khoder et al. 2022).

Moreover, whole-body exposure to a single dose of gamma IR (5 Gy) has significantly increased the level of TSH and reduced the level of serum T3 and T4 compared to the PTU-induced hypothyroidism group alone. These results were in agreement with Zhai et al. (2017) who verified that IR induced hypothyroidism. Misa-Agustiño et al. (2015) attributed IR-induced hypothyroidism to the massive damage and hypertrophy of the follicles together with its dysfunction subsequent to ROS production. In harmony with our results, Abdel-Aziz (2018) revealed that treating hypothyroid rats with MEE significantly decreased the serum TSH levels and increased the serum T3 and T4 levels. Moreover, using caffeic acid which is one of the active constituents of MEE abolished hypothyroidism in rats owing to its thyro-stimulatory potential as well as increasing the circulating levels of T3 through the mono-deiodination of T4 (Kar et al. 2022).

Thyroid hormones influence the balance between the antioxidant defense system and oxidative stress (Mancini et al. 2016). Both states of thyroid dysfunction (hyperthyroidism and hypothyroidism) promote oxidative damage in brain tissues displayed by elevated levels of MDA and NO concomitant with depletion of the enzymatic (SOD, CAT, and GPx) and non-enzymatic (GSH) antioxidants leading to brain damage (Ahmed et al. 2012; Farrokhi et al. 2014). Similarly, the present data revealed that in brain tissues, both hypothyroidism and radiation impaired the redox status and resulted in a state of oxidative stress, characterized by a marked decline in the antioxidant defense system (SOD, CAT, GSH, and GPx) accompanied by significantly increased levels of lipid peroxidation (MDA) as well as NO levels. Based on the excess content of polyunsaturated fatty acids, high oxygen consumption, and low antioxidant levels, the brain is particularly vulnerable to oxidative stress and ROS (Cheignon et al. 2018).

The present study showed a significant increase in antioxidant levels coupled with a remarkable reduction in both MDA and NO levels after MEE treatment. Farahi et al. (2012) reported that *Melissa officinalis* reduced lipid peroxidation owing to its antioxidant potential. Furthermore, Kamdem et al. (2013) reverted the powerful antioxidant effect of the MEE to higher content of phenolic (flavonoids) which boosts the scavenging of NO and free radicals together with hindering free radical chain reactions that propagate oxidative stress status, electron-donation ability, and chelation activity. Additionally, López et al. (2009) indicated that *Melissa officinalis* alleviated oxidative stress status in the neurons thus neuroprotective effect.

Regarding the vital role of thyroid hormones in brain development, it was found that hypothyroidism was associated with impaired memory and loss of cognitive and neurological functions and finally neuronal death (Liu and Brent 2018; Bernal 2022). Elbakry et al. (2022) observed that oxidative stress promoted the accumulation of misfolded proteins (amyloid-β and tau) in the brain tissues leading to neurodegeneration. Interestingly, it was reported that thyroid hormones affect the expression of APP; meanwhile, their deficiency was accompanied by a significantly upregulated expression of the APP mRNA (Liu and Brent 2018) which in turn led to the accumulation of Aβ and tau protein and eventually brain degeneration (Lewis et al. 2001). Furthermore, alternative splicing of the MAPT mRNA during translation or posttranslational resulted in its maturation and formation of tau protein, one of the main causes of neurodegeneration (Chang et al. 2021). Besides, Olczak et al. (2017) observed elevated levels of MAPT in brain injury and neurodegeneration diseases. On the contrary, Ozarowski et al. (2016) showed that *Melissa officinalis* through summation of its bioactive components diminished Aβ accumulation and alleviated cellular toxicity in PC12 cells.

Impaired redox status concomitant with sustained ER stress, as well as a plethora of the misfolded protein aggregates relevant to hypothyroidism, provoked cascades of neuronal death (Blas-Valdivia et al. 2021; Shi et al. 2022) that was obviously seen by the overexpression of the apoptotic markers (CHOP, Bax, and caspase-12) along with the suppression of the anti-apoptotic Bcl2 protein in the brain of the hypothyroid rats. Activation of PERK- eIF2α-ATF4 during the ER stress evoked CHOP activation and apoptosis (Han et al. 2021). CHOP mediates ER stress-induced apoptosis through modulation of the downstream pro-apoptotic proteins (BCL2 family) and caspases particularly caspase-12 which is found in the ER outer membrane and promoted ER stress-mediated apoptosis by activation of the mitochondrial caspases (3 and 9) (Han et al. 2021). However, hampering and suppression of the ER stress pathway (PERK- eIF2α-ATF4-CHOP) diminished the misfolded proteins (amyloidogenesis) (Shi et al. 2022) and attenuated the elicited apoptosis (Liu et al. 2021; Xu et al. 2021). Consequently, restoring ER homeostasis minimizes neuronal degeneration and brain damage and promotes their recovery. Previous studies attributed the neuroprotective effect of the *Melissa officinalis* to the alleviation of the oxidative stress and inhibition of apoptosis (Hassanzadeh et al. 2011; Hosseini et al. 2015).

Additionally, the abundance of polyphenolic compounds in the extract of *Melissa officinalis* activates vitagenes which are a group of genes involved in maintaining cellular homeostasis during stressful conditions. Vitagenes include heat shock proteins (Hsp), HO-1, GSH, thioredoxin, and sirtuin (Calabrese et al. 2009). These activated vitagenes counteract proteotoxic stress by maintaining protein homeostasis (synthesis, folding, and degradation), besides ameliorating cellular redox balance via modulation of oxidative stress and scavenging of free radicals (Siracusa et al. 2020). Moreover, the protective effect of vitagens against oxidative stress-induced neurodegenerative diseases was attributed to the inhibition of neuronal apoptosis subsequent to the upregulation of HO-1 and Bcl2 levels (Calabrese et al. 2010; Cornelius et al. 2013).

In conclusion, the current study revealed that *Melissa officinalis* extract ameliorates the induced hypothyroidism as well as the associated brain damage by enhancing the cellular redox balance, alleviating the neurotoxicity through abrogating the misfolded protein aggregates precursors (APP & MAPT). Moreover, MEE impedes brain apoptosis by the inhibition of the ER stress pathway (PERK-eIF2α-ATF4-CHOP) and the apoptotic mediators (Bax and caspase-12) along with activation of the anti-apoptotic protein (Bcl2). Collectively, MEE maintains ER and protein homeostasis, ameliorates brain structure and function, and prevents neuronal apoptosis. Hence, it could confer the neuroprotective potential of MEE against hypothyroidism-induced brain damage.

## Data Availability

The raw data supporting the conclusion of this article will be made available by the authors without undue reservation.

## Declarations

### Limitations and future direction of the study

The limitations of the present study, including the small number of rats and a relatively short period of follow-up, should be taken into account and indicate the need for further research.

## References

[CR1] Abdel-Aziz M (2018). Study on the therapeutic potential of Melissa officinalis (Lemon Balm) on male Wistar albino rats with induced-hypothyroidism. Al-Azhar J Pharm Sci.

[CR2] Abo-Zaid OA, Moawed FS, Ismail ES, Ahmed ESA (2023). β-Sitosterol mitigates hepatocyte apoptosis by inhibiting endoplasmic reticulum stress in thioacetamide-induced hepatic injury in γ-irradiated rats. Food Chem Toxicol.

[CR3] Ahmed OM, Ahmed RG, El-Gareib AW, El-Bakry AM, Abd El-Tawab SM (2012). Effects of experimentally induced maternal hypothyroidism and hyperthyroidism on the development of rat offspring: II-the developmental pattern of neurons in relation to oxidative stress and antioxidant defense system. Int J Dev Neurosci.

[CR4] Alva-Sánchez C, Becerril A, Anguiano B, Aceves C, Pacheco-Rosado J (2009). Participation of NMDA-glutamatergic receptors in hippocampal neuronal damage caused by adult-onset hypothyroidism. Neurosci Lett.

[CR5] Alzoubi KH, Gerges NZ, Aleisa AM, Alkadhi KA (2009). Levothyroxin restores hypothyroidism-induced impairment of hippocampus-dependent learning and memory: behavioral, electrophysiological, and molecular studies. Hippocampus.

[CR6] Ashraf H, Heydari M, Shams M, Zarshenas MM, Tavakoli A, Sayadi M (2022). Efficacy of ginger supplementation in relieving persistent hypothyroid symptoms in patients with controlled primary hypothyroidism: a pilot randomized, double-blind, placebo-controlled clinical trial. Evid Based Complement Alternat Med.

[CR7] Bancroft JD, Gamble M (2013). Theory and practice of histological techniques.

[CR8] Bernal J, Feingold KR, Anawalt B, Blackman MR (2022). Thyroid hormones in brain development and function. [Updated 2022 Jan 14]. Endotext [Internet].

[CR9] Blas-Valdivia V, Franco-Colín M, Rojas-Franco P, Chao-Vazquez A, Cano-Europa E (2021). Gallic acid prevents the oxidative and endoplasmic reticulum stresses in the hippocampus of adult-onset hypothyroid rats. Front Pharmacol.

[CR10] Calabrese V, Cornelius C, Dinkova-Kostova AT, Calabrese EJ (2009). Vitagenes, cellular stress response, and acetylcarnitine: relevance to hormesis. Biofactors.

[CR11] Calabrese V, Cornelius C, Dinkova-Kostova AT, Calabrese EJ, Mattson MP (2010). Cellular stress responses, the hormesis paradigm, and vitagenes: novel targets for therapeutic intervention in neurodegenerative disorders. Antioxid Redox Signal.

[CR12] Cervelli T, Basta G, Del Turco S, Patel VB, Preedy VR (2021). Chapter 30 - Effects of antioxidant nutrients on ionizing radiation-induced oxidative stress. Toxicology.

[CR13] Chaker L, Razvi S, Bensenor IM (2022). Hypothyroidism. Nat Rev Dis Primers.

[CR14] Chang CW, Shao E, Mucke L (2021). Tau: enabler of diverse brain disorders and target of rapidly evolving therapeutic strategies. Science.

[CR15] Cheignon C, Tomas M, Bonnefont-Rousselot D, Faller P, Hureau C, Collin F (2018). Oxidative stress and the amyloid beta peptide in Alzheimer’s disease. Redox. Biol.

[CR16] Chen X, Cubillos-Ruiz JR (2021). Endoplasmic reticulum stress signals in the tumour and its microenvironment. Nat Rev Cancer.

[CR17] Cioffi F, Giacco A, Goglia F, Silvestri E (2022). Bioenergetic aspects of mitochondrial actions of thyroid hormones. Cells.

[CR18] Cornelius C, Perrotta R, Graziano A, Calabrese EJ, Calabrese V (2013). Stress responses, vitagenes and hormesis as critical determinants in aging and longevity: mitochondria as a “chi”. Immun Ageing.

[CR19] De Vitis C, Capalbo C, Torsello A, Napoli C, Salvati V, Loffredo C, Blandino G, Piaggio G, Auciello FR, Pelliccia F, Salerno G, Simmaco M, Di Magno L, Canettieri G, Coluzzi F, Mancini R, Rocco M, Sciacchitano S (2022). Opposite effect of thyroid hormones on oxidative stress and on mitochondrial respiration in COVID-19 patients. Antioxidants (Basel).

[CR20] Ebrahim R (2020). Effect of Spirulina platensis against thyroid disorders associated with liver dysfunction and dyslipidaemia in irradiated rats. Egypt J Radiat Appl.

[CR21] Elbakry MMM, Mansour SZ, Helal H, Ahmed ESA (2022). Nattokinase attenuates bisphenol A or gamma irradiation-mediated hepatic and neural toxicity by activation of Nrf2 and suppression of inflammatory mediators in rats. Environ Sci Pollut Res Int.

[CR22] Farahi A, Kasiri M, Sudagar M, Soleimani Iraei M, Zorriehzahra S (2012). Effect of dietary supplementation of Melissa officinalis and Aloe vera on hematological traits, lipid oxidation of carcass and performance in rainbow trout (Oncorhynchus mykiss). Online J Anim Feed Res.

[CR23] Farrokhi E, Hosseini M, Beheshti F, Vafaee F, Hadjzadeh MA, Dastgheib SS (2014). Brain tissues oxidative damage as a possible mechanism of deleterious effects of propylthiouracil- induced hypothyroidism on learning and memory in neonatal and juvenile growth in rats. Basic Clin Neurosci.

[CR24] Fumarola A, Di Fiore A, Dainelli M, Grani G, Calvanese A (2010). Medical treatment of hyperthyroidism: state of the art. Exp Clin Endocrinol Diabetes.

[CR25] Głombik K, Detka J, Bobula B, Bąk J, Kusek M, Tokarski K, Budziszewska B (2021). Contribution of hypothyroidism to cognitive impairment and hippocampal synaptic plasticity regulation in an animal model of depression. Int J Mol Sci.

[CR26] Han Y, Yuan M, Guo YS, Shen XY, Gao ZK, Bi X (2021). Mechanism of endoplasmic reticulum stress in cerebral ischemia. Front Cell Neurosci.

[CR27] Hashem H, Saad S (2020). Comparative study of the effect of experimentally induced hyperthyroidism and hypothyroidism on the parotid gland in adult male albino rats. Egypt J Histol.

[CR28] Hassanzadeh G, Pasbakhsh P, Akbari M, Shokri S, Ghahremani M, Amin G, Kashani I, Azami Tameh A (2011). Neuroprotective properties of Melissa officinalis L. extract against ecstasy-induced neurotoxicity. Cell J.

[CR29] Hetz C, Papa FR (2018). The unfolded protein response and cell fate control. Mol Cell.

[CR30] Hosseini R, Kaka G, Joghataei MT, Hooshmandi M, Sadraie SH (2015). Neuroprotective effect of Melissa officinalis in animal model of spinal cord injury. Med Aromat Plants.

[CR31] Issa N, El-Sherif N (2017). Effect of ginseng on the testis of subclinical hypothyroidism model in adult male Albino rat. Austin J Anat.

[CR32] Kamdem JP, Adeniran A, Boligon AA, Klimaczewski CV, Elekofehinti OO (2013). Antioxidant activity, genotoxicity and cytotoxicity evaluation of lemon balm (melissaofficinalis l.) ethanolic extract: its potential role in neuroprotection. Ind Crops Prod.

[CR33] Kar A, Panda S, Singh M, Biswas S (2022). Regulation of PTU-induced hypothyroidism in rats by caffeic acid primarily by activating thyrotropin receptors and by inhibiting oxidative stress. Phytomedicine Plus.

[CR34] Kato H, Liu Y, Araki T, Kogure K (1991). Temporal profile of the effects of pretreatment with brief cerebral ischemia on the neuronal damage following secondary ischemic insult in the gerbil: cumulative damage and protective effects. Brain Res.

[CR35] Khoder NM, Sawie HG, Sharada HM, Hosny EN, Khadrawy YA, Abdulla MS (2022). Metformin and alpha lipoic acid ameliorate hypothyroidism and its complications in adult male rats. J Diabetes Metab Disord.

[CR36] Lewis J (2001). Enhanced neurofibrillary degeneration in transgenic mice expressing mutant tau and APP. Science.

[CR37] Liu X, Chen Y, Wang H, Wei Y, Yuan Y, Zhou Q (2021). Microglia-derived IL-1beta promoted neuronal apoptosis through ER stress-mediated signaling pathway PERK/eIF2alpha/ATF4/CHOP upon arsenic exposure. J Hazard Mater.

[CR38] Liu YY, Brent GA (2018). Thyroid hormone and the brain: mechanisms of action in development and role in protection and promotion of recovery after brain injury. Pharmacol Ther.

[CR39] Livak KJ, Schmittgen TD (2001). Analysis of relative gene expression data using real-time quantitative PCR and the 2− ΔΔCT method. Methods.

[CR40] López V, Martín S, Gómez-Serranillos MP (2009). (2009) Neuroprotective and neurological properties of Melissa officinalis. Neurochem Res.

[CR41] Mancini A, Di Segni C, Raimondo S, Olivieri G, Silvestrini A, Meucci E, Currò D (2016). Thyroid hormones, oxidative stress, and inflammation. Mediators Inflamm.

[CR42] Mannaa FA, Abdel-Wahhab KG, Hassan LK, Taher RF, Morsy FA, Fadl NN (2021). Influence of Melissa officinalis methanolic extract on hyperthyroidism in a rat model. Egypt Pharm J.

[CR43] Mao J, Hu Y, Ruan L, Ji Y, Lou Z (2019). Role of endoplasmic reticulum stress in depression (Review). Mol Med Rep.

[CR44] Mincer DL, Jialal I (2022). Hashimoto thyroiditis. StatPearls [Internet].

[CR45] Miraj S, Rafieian-Kopaei KS (2017). *Melissa officinalis* L A review study with an antioxidant perspective. J Evid Based Complementary Altern Med.

[CR46] Misa-Agustiño MJ, Jorge-Mora T, Jorge-Barreiro FJ, Suarez-Quintanilla J, Moreno-Piquero E, Ares-Pena FJ, López-Martín E (2015). Exposure to non-ionizing radiation provokes changes in rat thyroid morphology and expression of HSP-90. Exp Biol Med (Maywood).

[CR47] Muppidi V, Meegada SR, Rehman A (2023). Secondary hyperparathyroidism. 2023 Feb 12. StatPearls [Internet].

[CR48] Nagayama Y (2018). Radiation-related thyroid autoimmunity and dysfunction. J Radiat Res (Tokyo).

[CR49] Olczak M, Niderla-Bielińska J, Kwiatkowska M, Samojłowicz D, Tarka S, Wierzba-Bobrowicz T (2017). Tau protein (MAPT) as a possible biochemical marker of traumatic brain injury in postmortem examination. Forensic Sci Int.

[CR50] Ozarowski M, Mikolajczak PL, Piasecka A, Kachlicki P, Kujawski R (2016). Influence of the Melissa officinalis leaf extract on long-term memory in scopolamine animal model with assessment of mechanism of action. Evid Based Complement Alternat Med.

[CR51] Phillips MJ, Voeltz GK (2016). Structure and function of ER membrane contact sites with other organelles. Nat Rev Mol Cell Biol.

[CR52] Samuels MH, Bernstein LJ (2022). Brain fog in hypothyroidism: what is it, how is it measured, and what can be done about it. Thyroid.

[CR53] Sandow JJ, Dorstyn L, O’Reilly LA, Tailler M, Kumar S, Strasser A (2014). ER stress does not cause upregulation and activation of caspase-2 to initiate apoptosis. Cell Death Differ.

[CR54] Shi M, Chai Y, Zhang J, Chen X (2022). Endoplasmic reticulum stress-associated neuronal death and innate immune response in neurological diseases. Front Immunol.

[CR55] Sipos S, Moacă EA, Pavel IZ, Avram Ş, Crețu OM, Coricovac D (2021). *Melissa officinalis* L. aqueous extract exerts antioxidant and antiangiogenic effects and improves physiological skin parameters. Molecules.

[CR56] Siracusa R, Scuto M, Fusco R, Trovato A, Ontario ML, Crea R, Di Paola R, Cuzzocrea S, Calabrese V (2020). Anti-inflammatory and anti-oxidant activity of Hidrox^®^ in rotenone-induced Parkinson’s disease in mice. Antioxidants (Basel).

[CR57] Talhada D, Santos CRA, Gonçalves I, Ruscher K (2019). Thyroid hormones in the brain and their impact in recovery mechanisms after stroke. Front Neurol.

[CR58] Torres-Manzo AP, Franco-Colín M, Blas-Valdivia V, Pineda-Reynoso M, Cano-Europa E (2018). Hypothyroidism causes endoplasmic reticulum stress in adult rat hippocampus: a mechanism associated with hippocampal damage. Oxid Med Cell Longev.

[CR59] Wahman LF, Abd Rabo MM, Elgoly AHM, Yousef MH (2020). Potential role of plants Hordeum vulgare L. and Panax ginseng L. in resolving the fertility disorders and stress-induced oxidative stress arises from hypothyroidism in adult female rats. Plant Stress Physiology.

[CR60] Xu L, Bi Y, Xu Y, Wu Y, Du X, Mou Y (2021). Suppression of CHOP reduces neuronal apoptosis and rescues cognitive impairment induced by intermittent hypoxia by inhibiting Bax and Bak activation. Neural Plast.

[CR61] Zhai R-p, Kong F-f, Du C-r, Hu C-s, Ying H-m (2017). Radiation-induced hypothyroidism after IMRT for nasopharyngeal carcinoma: clinical and dosimetric predictors in a prospective cohort study. Oral Oncol.

